# Volatile Compounds of Selected Raw and Cooked *Brassica* Vegetables

**DOI:** 10.3390/molecules24030391

**Published:** 2019-01-22

**Authors:** Martyna N. Wieczorek, Henryk H. Jeleń

**Affiliations:** Faculty of Food Science and Nutrition, Poznań University of Life Sciences, Wojska Polskiego 31, 60-624 Poznań, Poland; martyna.wieczorek@up.poznan.pl

**Keywords:** *Brassica* vegetables, volatile compounds, cooking, GC×GC-ToFMS

## Abstract

*Brassica* vegetables are a significant component of the human diet and their popularity is systematically increasing. The interest in plants from this group is growing because of numerous reports focused on their pro-health properties. However, some consumers are not enthusiastic about these vegetables because of their specific bitter taste and sharp, sulfurous aroma. In this study, the volatile composition of 15 *Brassica* cultivars (five Brussels sprouts, four kohlrabi, three cauliflower and three broccoli), both raw and cooked, was analyzed by solid phase microextraction and comprehensive two-dimensional gas chromatography with time of flight mass spectrometry (SPME-GC×GC-ToFMS). Differences were found between the analyzed vegetables, as well as different cultivars of the same vegetable. Moreover, the influence of cooking on the composition of volatile compounds was evaluated. All the vegetables were frozen before analyses, which is why the impact of this process on the volatile organic compounds (VOCs) was included. The most abundant groups of compounds were sulfur components (including bioactive isothiocyanates), nitriles, aldehydes and alcohols. Cooking in general caused a decrease in the abundance of main volatiles. However, the amount of bioactive isothiocyanates increased in most cultivars after cooking. The effect of freezing on the volatile fraction was presented based on the Brussels sprout cultivars. Most of the changes were closely related to the activity of the lipoxygenase (LOX) pathway enzymes. These are characterized by a marked reduction in alcohol contents and an increment in aldehyde contents. Moreover, important changes were noted in the concentrations of bioactive components, e.g., isothiocyanates. This research included a large set of samples consisting of many cultivars of each analyzed vegetable, which is why it provides a considerable body of general information concerning volatiles in *Brassica* vegetables.

## 1. Introduction

Broccoli, Brussels sprouts, cauliflower and kohlrabi are members of the Brassicaceae family, which is widely cultivated throughout the world. Some consumers reject *Brassica* vegetables due to their characteristic flavor, which is genetically related [1]. The consumption of Brassicaceae is high; however, it is mostly in the cooked form because of significant changes in flavor. Thermal treatment makes vegetables easily digestible and more acceptable for consumers. Nevertheless, some of them, e.g., broccoli, cauliflower or kohlrabi, are sometimes eaten raw, usually as an ingredient in salads. Thermal treatment affects the concentration of many beneficial compounds in vegetables, such as vitamins or phenolic compounds [2], while at the same time significantly changing the flavor profile of these vegetables. Many *Brassica* vegetables are sold as frozen florets or cubes, often found as ingredients in vegetable mixes, thus ensuring their supply year-round in a convenient form. Raw vegetables are rich in aromatic compounds, which usually are produced as a result of enzymatic reactions. Their storage in the frozen form can significantly alter biochemical reactions occurring in vegetables, thus influencing flavor also after cooking. However, there are practically no studies on the comparison of volatile compound profiles of frozen, fresh and cooked *Brassica* vegetables. In the case of the analyzed plants, the specific interest focused on bioactive molecules is particularly high and they have been extensively studied recently, mainly due to the presence of isothiocyanates. These are the products of enzymatic hydrolysis of glucosinolates and their beneficial influence on human health has been widely examined [3,4,5,6,7,8].

A wide array of analytical techniques have been used to analyze volatile compounds in plants, including fruit and vegetables. *Brassica* volatiles have been collected using simultaneous distillation-extraction (SDE) [9,10], sorbent (Tenax) trapping [11], solvent extraction [12], as well as solid phase microextraction (SPME) [13,14]. The latter is the most widely applied solvent-free isolation method for plant materials and food flavors, thanks to its sensitivity, preconcentration abilities, ruggedness, potential automation and ease of use [15,16].

The aim of the present study was to evaluate differences in the profiles of volatile compounds in fresh and cooked *Brassica* vegetables. Thanks to the results obtained for 15 different cultivars of kohlrabi, broccoli, cauliflower and Brussels sprouts, generalized conclusions may be drawn in terms of changes in volatile compounds in the main representatives of *Brassica* vegetables. The volatile compounds were analyzed using solid phase microextraction and comprehensive two-dimensional gas chromatography with time of flight mass spectrometry (SPME-GC×GC-ToFMS).

## 2. Results

### 2.1. Identification of Volatiles

To compare the profiles of volatiles for the investigated vegetables, the compounds were isolated using headspace analysis, where the volatiles were adsorbed using SPME fiber and then separated by comprehensive two-dimensional chromatography-mass spectrometry (HS-SPME-GC×GC-ToFMS). This approach allows for the specific separation and (tentative in this case) identification of volatile compounds, thus, it is widely used in volatilome fingerprinting and profiling [17,18]. As the peak capacity of GC×GC systems can reach 10,000, the number of identified compounds is usually very high. GC×GC-ToFMS provides plenty of data, for this reason extracting useful information is often a very challenging task. For our work, only the most abundant compounds with a relatively high signal to noise ratio (S/N > 250), high similarity and retention indexes comparable to literature data were taken into consideration and the comparison was based on the peak area values and the peak area percentage. This serves the assumed comparative purposes well; however, it has to be remembered that SPME is a partition coefficient based extraction method and the area percentage may be different from the data provided by exhaustive extraction methods.

**Figure 1:**
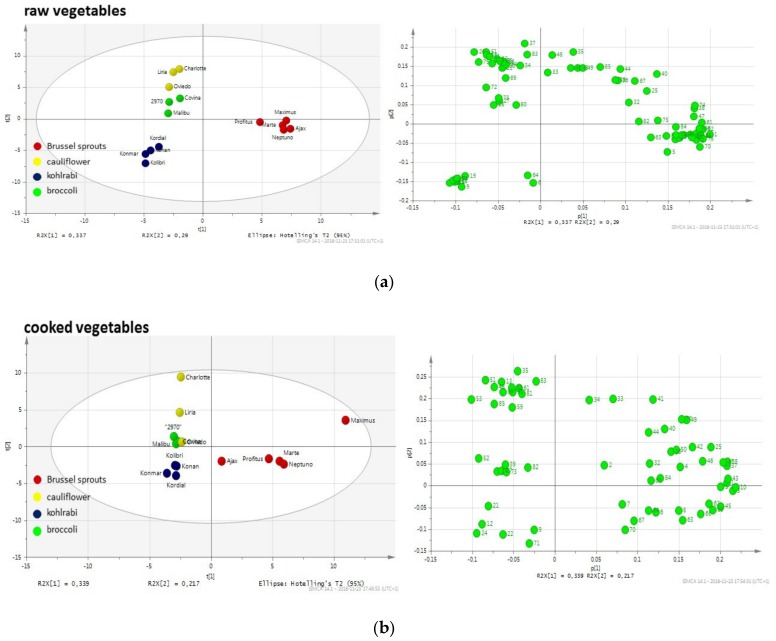
Principal component analysis (PCA) graphs of volatiles extracted from the raw (**a**) and cooked (**b**) *Brassica* vegetables investigated. Numbers on loadings plot represent compounds characteristic for analyzed vegetables. Compound numbering is uniform with data provided in the Supplementary Table S5.

**Figure 2:**
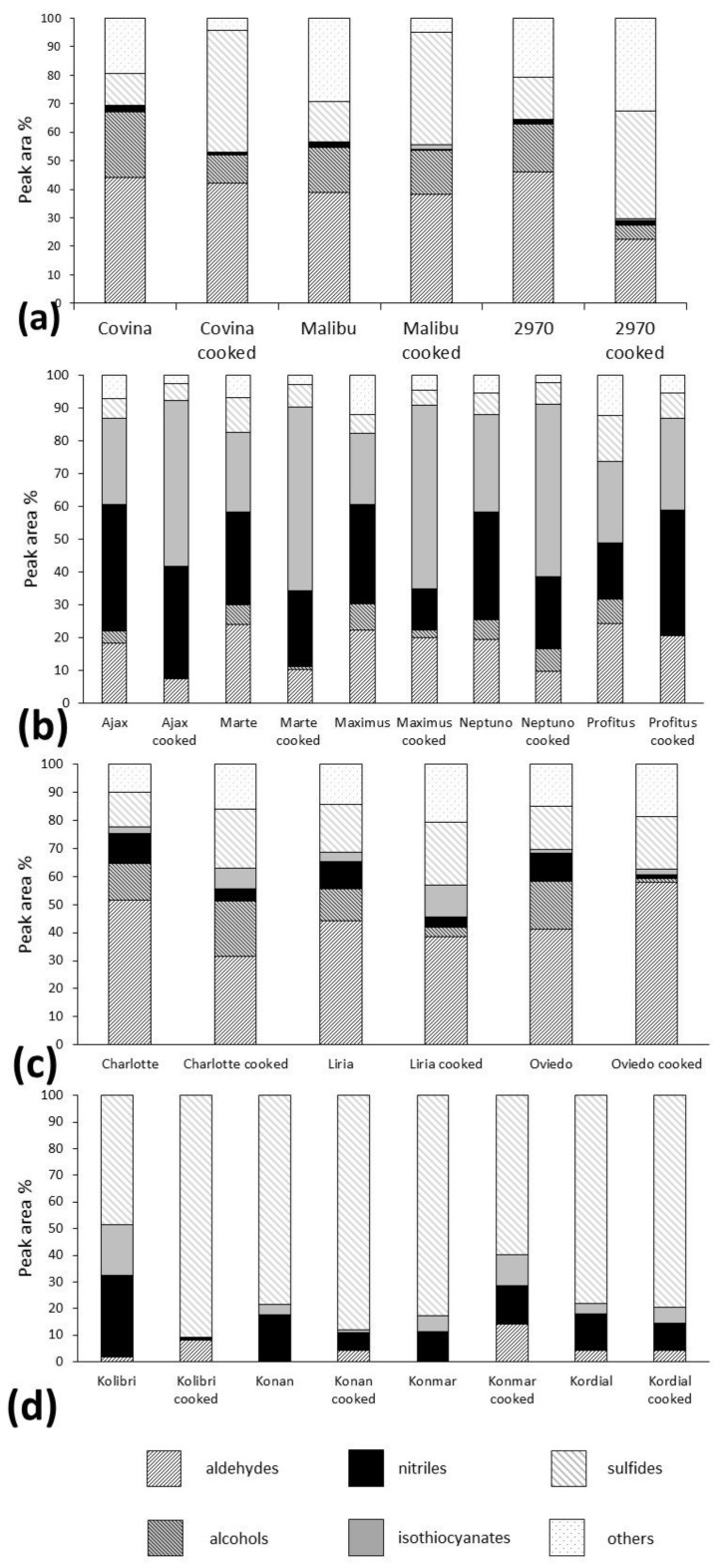
Percentage contents of isothiocyanates, alcohols, aldehydes, other sulfur compounds, nitriles, others (miscellaneous) in all analyzed samples. Broccoli (**a**): Covina, Malibu, “2970”; Brussels sprouts (**b**): Ajax, Marte, Maximus, Neptuno, Profitus; Cauliflower (**c**): Charlotte, Liria, Oviedo; Kohlrabi (**d**): Kolibri, Konan, Konmar, Kordial.

Figure 1 shows the differentiation between various *Brassica* vegetables using principal component analysis (PCA) based on the profiles of volatile compounds. Each point represents a mean of three replicates analyzed for a given cultivar. One can observe obvious differences in the profiles of particular vegetables, both raw and cooked. However, there are also significant differences between particular cultivars of some investigated vegetables, which is especially noteworthy for raw broccoli and Brussels sprouts. Examination of the PCA graphs shows a similarity between cauliflower and broccoli volatiles in raw vegetables, despite the between-cultivar differences for broccoli, while kohlrabi and Brussels sprouts differ substantially in terms of their volatile compound profiles. When the data are compared for cooked vegetables, the similarities between cauliflower and broccoli can still be observed (close distance between clusters), whereas the other vegetables form distant clusters. Interestingly, after cooking, the differences between cultivars are much less pronounced. This would indicate the more uniform profile of the main volatiles. A detailed list of main compounds for all the examined cultivars is provided in Tables S1–S4 in the supplementary files. The compounds corresponding to the number in the PCA loading plots are presented in Table S5 in the supplementary files. As can be observed in the tables, the compounds can be classified into several main groups: Aldehydes (with alkanals, 2-alkenals and 2,4-alcadienals), alcohols (mainly unsaturated), isothiocyanates (mainly aliphatic, although also aromatic), other sulfur compounds (mainly sulfides and thiophenes) and nitriles. To a certain extent, the classification of volatile compounds into these classes is also associated with the sensory properties and acceptance of these compounds. Some of the identified aldehydes and alcohols may contribute to the “green”, “sweet”, “fatty”, “soup” and other notes in the vegetable aroma. Isothiocyanates are products of the enzymatic hydrolysis of glucosinolates, which are widely investigated because of their beneficial effect on human health. However, they are also partially responsible for the specific pungent notes. Nitriles, which are an abundant group in all analyzed samples, are also found as degradation products of glucosinolates, instead of isothiocyatates [1]. Other sulfur components include mainly products of the cysteine pathway, which are sulfides. All the detected sulfides, i.e., disulfides, trisulfides, tetrasulfides, pentasulfides, and others have been collected under the general name of “sulfides”, simply because of their labile nature and mutual transposition. To facilitate an easy visual comparison of raw and cooked cultivars, Figure 2 shows the main groups of the detected compounds in the four examined vegetables.

#### 2.1.1. Concentrations of Isothiocyanates and Nitriles in Raw and Cooked *Brassica* Vegetables

Very small amounts of isothiocyanates were detected in all of the raw broccoli cultivars (<2% in all samples). Additionally, the nitrile levels were relatively low (<5%), which confirms very low concentrations of myrosinase hydrolysis products in the gas phase. The diversity of isothiocyanates was also very limited, barely four different isothiocyanates were identified using GC×GC. Both 2-methylbutyl isothiocyanate and isobutyl isothiocyanate were found in the cultivars Malibu and “2970”, while cyclopentyl isothiocyanate and n-hexyl isothiocyanate were present in all the analyzed broccoli samples. The composition of isothiocyanates was different in cooked vegetables, as propane, 1-isothiocyanato-, butane, 1-isothiocyanato-, 1-butene, 4-isothiocyanato- and benzene, (2-isothiocyanatoethyl)- were detected in all the analyzed cooked vegetables, instead of most isothiocyanates present in raw vegetables. The most interesting result was found in the cooked cultivar “2970”, where all the described isothiocyanates were present, along with a significant amount of allyl isothiocyanate (which was not present in the raw broccoli). This unexpected finding was connected with the absence of sulforaphane, one of the most explored isothiocyanates present in broccoli. Because of the health benefits of sulforaphane [6], interest in this component concentration is particularly high. Solvent assistant flavor evaporation (SAFE) extraction was performed for one broccoli cultivar to check if the sulforaphane was present in the sample. In the SAFE extract, analyzed by GC×GC-ToFMS, sulforaphane and also sulforaphane nitrile were present as glucoraphanin degradation products, although in very small concentrations. The absence of sulforaphane in the SPME extract may either be due to the labile nature of this component [19], or more probably, due to its small K value (partition coefficient sample-headspace-fiber). SAFE is an exhaustive extraction type technique, therefore other/more components can be identified.

The concentration and diversity of isothiocyanates was much greater in the cooked broccoli. The amounts were almost two times higher than in the raw vegetables. It was reported that myrosinase in the broccoli matrix is inactivated after 20 min at 60 °C [20]. No reports have been found in available literature on the stability of nitrile specific proteins. Generally, the total amount of glucosinolate degradation products decreased, especially the concentration of nitriles. Cooking caused a change in the amount of isothiocyanates, which is beneficial from the consumer’s point of view.

In cauliflower, the major isothiocyanate was allyl isothiocyanate, representing 2–4% of the total volatile fraction, resulting from the high concentration of sinigrin, its precursor, in cauliflowers [12]. Allyl isothiocyanates are some of most frequently studied isothiocyanates, with many publications confirming their anticancer activity in both cultured cancer cells and animal models. Moreover, the bioavailability of this compound is very high, as almost 90% of oral intake is absorbed [21]. Other isothiocyanates were also present, although in much lower concentrations, usually less than 1%. In the Lira and Charlotte cultivars, somewhat higher amounts of isothiocyanates were recorded in the cooked vegetables compared with the raw vegetables, while in the Oviedo cultivar the situation is opposite. An abundant group in all raw cauliflower cultivars was composed of nitriles, which constituted almost 10% in all analyzed samples. It proves again, that also in raw cauliflower after tissue disruption, nitriles are formed as the main products of glucosinolate hydrolysis. It suggests that after tissue disruption, the hydrolysis of glucosinolates yields nitriles instead of bioactive isothiocyanates. The concentration of 2-methylbutyl isothiocyanate was higher in the cooked vegetables in all analyzed varieties, whereas the allyl isothiocyanate contents were higher in the raw vegetables (except for the Liria cultivar). The total amount of isothiocyanates was higher in the cooked Lira and Charlotte cultivars than in the raw vegetables (Table S2). An opposite situation was observed in the Oviedo cultivar. The nitrile concentration was significantly smaller in all analyzed cooked cauliflower samples.

The third group of analyzed plants comprised four varieties of kohlrabi: Kolibri, Kordial, Konan and Konmar. The percentage concentration of isothiocyanates in the raw vegetables was unexpectedly varied, as it was 4.16% for Konan, 18.96% in Kolibri, 6.15% Konmar and 3.71% in Kordial. The content slightly exceeded that in cauliflower. This fact was also correlated with high nitrile concentrations (more than 10% in all cases). In cooked kohlrabies, this amount increased in the Konmar and Kordial cultivars. Benzene, (2-isothiocyanatoethyl)-, which is present in uncooked vegetables at significant levels, almost disappeared in the cooked kohlrabies. The increase in isothiocyanate concentrations (Table S3) in the Konmar and Kordial cultivars was mostly caused by the increase of the n-pentyl isothiocyanate peak area in those two cultivars. The peak area (i.e., amounts) of nitriles decreased significantly in all analyzed cooked kohlrabi varieties, compared to the raw varieties.

The uncooked Brussels sprout cultivars contained mostly nitriles in their volatile fractions. Isothiocyanates constituted the second most abundant group. In the cooked plants, the isothiocyanates accounted for more than 50% in the Maximus, Marte, Ajax and Neptuno cultivars. However, the percentage contents of isothiocyanates increased, while their summary peak area changed significantly only in the Maximus cultivar. In the case of nitriles, the summary peak area decreased almost two-fold when compared with the raw varieties. Only in the Profitus cultivar did the levels of nitriles remain practically unchanged.

**Figure 3:**
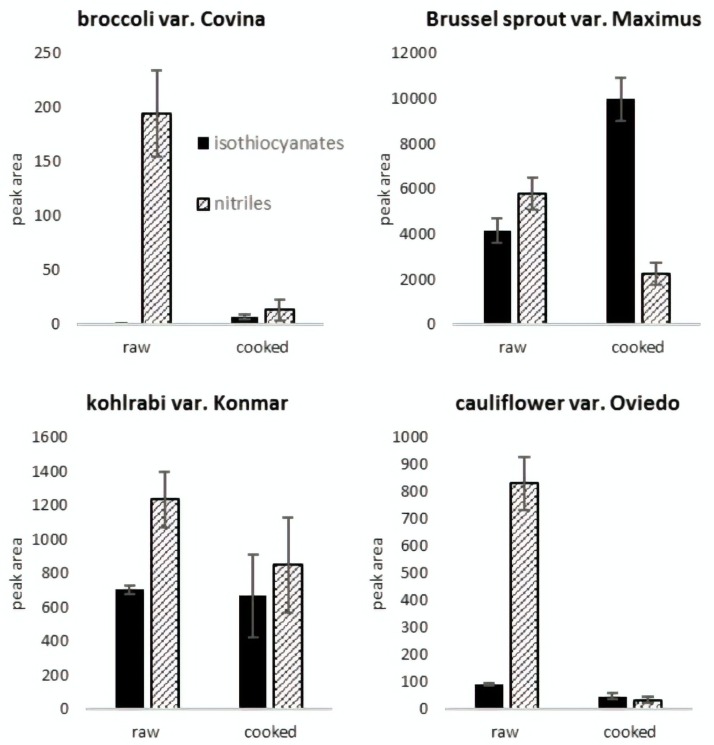
Proportions between isothiocyanates and nitriles in selected raw and cooked *Brassica* vegetables. The results are the mean of three replicates and the error bars show standard deviation. Amounts were expressed in total (sum of) peak areas.

The important issue described in this part of the study is related with the high concentration of nitriles in some of the analyzed samples. This fact suggests that after tissue disruption in raw, defrosted vegetables, the hydrolysis of glucosinolates occurs in favor of the formation of nitriles instead of bioactive isothiocyanates. This seems to be important from a biological point of view. This is because of the health-promoting nature of isothiocyanates, which are bioactive molecules that are known for having many positive effects on human health. These isothiocyanates are found as a minority in the volatile fraction with respect to nitriles. Based on the actual in vitro data, nitriles have less beneficial health potential [19,22,23], or even harmful effects on consumers [24]. In cauliflower, the percentage of nitriles was more than 10% and the percentage of isothiocyanates was about 3% in all samples, with a similar situation observed in broccoli. In the kohlrabi cultivars, the level of isothiocyanates was slightly higher; however, the nitrile concentration was still two times higher, and in the Konan cultivar it was even four times higher than that of the isothiocyanates. As presented, the enzymatic degradation of glucosinolates leads to the formation of isothiocyanates, and in some cases, to nitriles, as the main product. The presence of modifying proteins such as the epithiospecifier protein or the nitrile-specifier proteins results in the enzymatic degradation of glucosinolates being altered in favor of nitriles [8,25]. It is important to highlight here that all vegetables were frozen before analysis; the impact of freezing on the composition of volatiles is presented in the next part of the paper. The changes in isothiocyanates and nitriles induced by cooking are presented for selected cultivars in Figure 3. It illustrates the general decrease in nitriles caused by cooking and also the different behavior of isothiocyanates in these cultivars. Initially, research on glucosinolate degradation products was mainly focused on their toxic, antinutritive and goitrogenic properties. More recently, attention has shifted to investigations concerning their beneficial effects against various diseases. Most studies are focused on different *Brassica* vegetables and the detection of glucosinolates, which are biologically non-active molecules. Still, a higher glucosinolate content does not always guarantee an increment of desirable isothiocyanates after tissue mastication. The formation of beneficial isothiocyanates depends on a variety of factors, such as the activity of myrosinase and nitrile specific proteins or domestic processing. It is worth mentioning that even if glucosinolates are not degraded, their consumption is beneficial, since they can be hydrolyzed by a healthy intestinal microbiome [26]. Studies focusing on the health-promoting effects of isothiocyanates are numerous; however, they are mostly focused on sulforaphane [27], allyl isothiocyanate [21] and benzyl isothiocyanate [28], while as presented the qualitative diversity of isothiocynates is considerable.

#### 2.1.2. Other Sulfur Volatiles

Derivatives from the *S*-alk(en)yl-l-cysteine pathway compounds occurred very frequently in the volatile fraction in all analyzed vegetables. In all raw kohlrabi cultivars, sulfides formed the most abundant group of volatile components. They accounted for more than 50% of all the volatiles present in the analyzed kohlrabies (Figure 2). In the Konmar cultivar, it was almost 80%. Surprisingly, a small total percentage of sulfides was noted in raw Brussels sprouts cultivars, where “other sulfides” represented less than 15% in all analyzed samples. Numerously represented sulfides (di, tri-, tetra-) are shown in the supplementary tables. The average percentage content of sulfides was approximately 10% in all analyzed *Brassicaceae*. Taking into account the extremely low odor threshold of these compounds, which are detectable at levels as low as one part per trillion by the human nose [29], their crucial role in the *Brassicaceae* characteristic flavor formation seems to be obvious. The smell of all sulfides detected here is described as extremely unpleasant [29]. The comparison of sulfide concentrations in raw and cooked vegetables was inconclusive, because in broccoli, the amount of sulfides was higher in the cooked form than in the raw form, while in kohlrabi, cauliflower and Brussels sprouts, the concentration was usually higher in the cooked vegetable. Additionally, there were some exceptions for some cultivars, where the proportions were reversed.

#### 2.1.3. Aldehydes and Alcohols

All the analyzed cauliflower and broccoli cultivars contained high percentage levels of aldehydes. In broccoli, the dominating aldehydes were hexenal, 2-hexenal (more than 10% in all samples), 2,4-heptadienal and benzaldehyde. According to [29], those aldehydes contribute the “green” type aromas to the final broccoli fragrance. In the cauliflower cultivars, the content of aldehydes was even higher than in broccoli, with 2,4-heptadienal, 2-hexenal, hexanal, benzaldehyde, propanal and nonanal being dominant (found in the highest percentage levels). The presented profile of aldehydes in cauliflower was quite similar to that in broccoli. A marked decline in the diversity and percentage contents of aldehydes was observed in all kohlrabi varieties. In all of the analyzed cultivars, the level of aldehydes was below 5%. The magnitudes of the aldehyde peaks on the chromatograms were also relatively small. In comparison with cauliflower and broccoli, which contain respectively 22 and 16 different aldehydes, seven aldehydes were identified in kohlrabi after GC×GC analysis, seemingly to be a very small number. The most abundant aldehyde in all varieties was 2-butenal, with a flower-type odor [29]. In Brussels sprouts, the aldehyde percentage content was low; however, their peak areas and diversity were relatively high. Fifteen different aldehydes were identified by GC×GC, with the ratios of all aldehydes being similar and with benzaldehyde being the most abundant. Twelve different alcohols were also present in the Brussels sprouts chromatograms and their percentage contents were significantly higher than those of the aldehydes (upper 20%). Only seven different aldehydes were identified in the kohlrabi samples, at less than 5% in all samples. Despite the small amounts of aldehydes, which are mostly important for “green”, “sweet” and “nice” aromas, the intensity of “raw kohlrabi” and “green” odors detected by the panelists was relatively high. Alcohols were present in trace amounts, so they were disregarded in the percentage calculations. In cooked broccoli and cauliflower, the number of aldehydes was noticeably lower in the cooked vegetables, compared to raw varieties. In contrast, the number of aldehydes in the kohlrabi was significantly greater in the cooked vegetables. The percentage contents of different aldehydes in cooked kohlrabi varied between cultivars. In Brussels sprouts, the levels of the lipoxygenase (LOX) pathway products decreased in all cooked cultivars.

**Figure 4:**
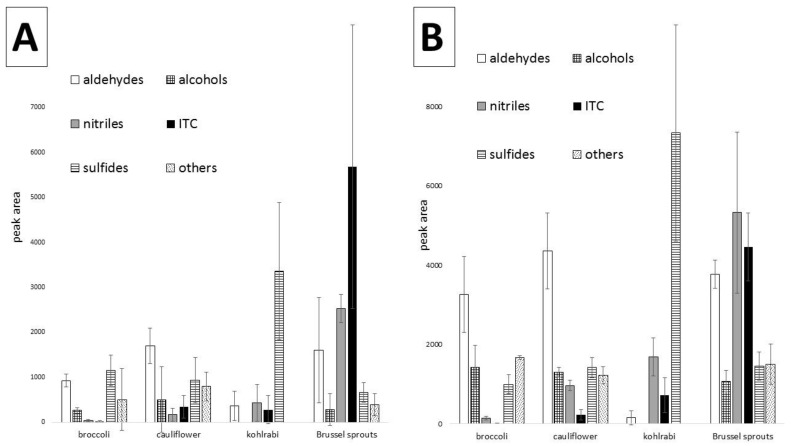
Mean values in different cultivars of analyzed raw (**A**) and cooked (**B**) vegetables. For broccoli, it is the mean value from three cultivars: Covina, Malibu and “2970”, for cauliflower, from three cultivars: Charlotte, Oviedo, Liria, for kohlrabi, from four cultivars: Kolibri, Konan, Konmar, Kordial and for Brussels sprouts, from five cultivars: Ajax, Marte, Maximus, Neptuno, Profitus. The results are the mean of three replicates for each cultivar (i.e., nine analyses for broccoli and cauliflower, 15 for Brussels sprouts and 12 for kohlrabi), error bars show standard deviation. Amounts were expressed in total (sum of) peak areas.

Figure 4 shows the peak areas of the main groups of volatile compounds investigated in the analyzed vegetables, both raw and cooked. Total peak areas were used to indicate the differences between particular vegetables, so it can be seen that aldehydes are dominant in broccoli and cauliflower, whereas in kohlrabi, sulfides constitute the most abundant fraction. Additionally, the dominance of nitriles and isothiocyanates in raw Brussels sprouts is easily noted. The error bars indicate differences caused by different cultivars taken for comparison, therefore they are in some instances relatively high. Similarly, the main fractions of the examined cooked vegetables can be compared, indicating an overall decrease in the amounts of volatiles caused by cooking, with some exceptions discussed above.

#### 2.1.4. Freezing-Thawing Effect on the Volatile Fraction

**Figure 5:**
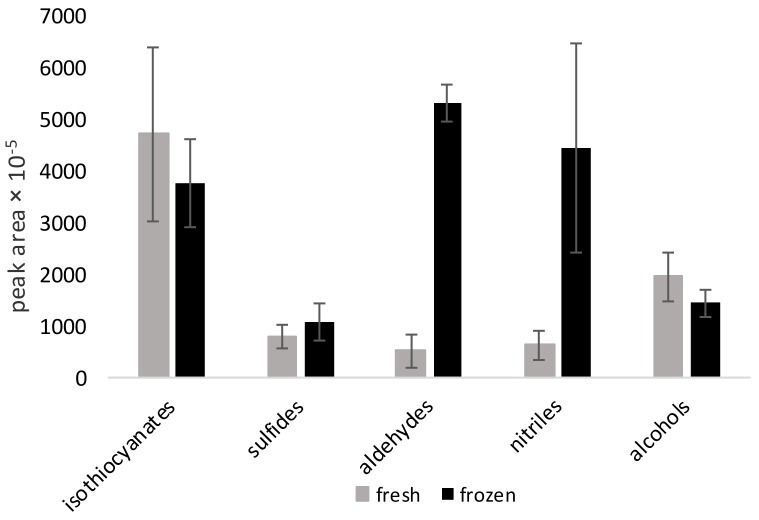
Mean values from all Brussels sprout cultivars (Ajax, Marte, Maximus, Neptuno and Profitus) for total peak areas of the main groups in raw non-frozen and frozen-thawed vegetables. The results are the mean of three replicates for each cultivar (i.e., 15 analyses), error bars show standard deviation. Amounts were expressed in total (sum of) peak areas.

Prior to cooking, all samples were stored at −20 °C, which is why the impact of freezing needed to be evaluated. The comparison of the volatile composition in raw, fresh and frozen-thawed vegetables was performed based on Brussels sprouts. Figure 5 shows a comparison of nitrile amounts in raw (fresh) and raw frozen-thawed vegetables for all 5 Brussels sprout cultivars. Moreover, the percentage contents of aldehydes was also higher than those of alcohols in the frozen vegetables, whereas the opposite situation was observed in fresh tissue. It is worth highlighting here that in the fresh product, the concentration of isothiocyanates was more than 50% of the total value, whereas in the frozen vegetables the amount was approximately 30%. The reason for the change in the isothiocyanate levels and an increase of nitrile contents is unknown at the moment. It may be connected with cell degradation during the freezing process, leading to changes in the activity/stability of enzymes responsible for the hydrolysis of glucosinolates.

## 3. Materials and Methods

### 3.1. Brassica Cultivars

Three cultivars of fresh broccoli (Covina, “2970”, Malibu), three cultivars of cauliflower (Charlotte, Oviedo, Liria), four of kohlrabi (Konmar, Kolibri, Konan, Kordial) and five cultivars of Brussels sprouts (Ajax, Maximus, Profitus, Naptuno, Marte) were used for analysis. They were harvested during the same 2016 autumn season: Broccoli, cauliflower and kohlrabi were harvested in September and the Brussels sprout cultivars were harvested in December. The vegetables were delivered to the laboratory within 24 h after harvest and stored at −20 °C until analysis (approximately 4 weeks).

### 3.2. Cooking Process

All vegetables were cooked in the same way. After washing and chopping, about 200 g of plant material was placed in 1 L of boiling pure water with 7 g of salt. Cooking times differed among vegetables. It was 5 min for the broccoli, 7 min for the cauliflower and 10 min for the kohlrabi and Brussels sprouts.

### 3.3. GC×GC-ToFMS Analysis

The volatiles of the *Brassica* vegetable samples were isolated using headspace solid phase microextraction (HS-SPME) and analyzed using SPME-GC×GC-TOFMS for the comparative, semiquantitive (peak area percentage) analysis only. The SPME fiber carboxen/polydimethylsiloxane (DVB/CAR/PDMS) was used for volatile extraction (Supelco, Bellefonte, PA, USA). The vegetables were cut into approximately 0.5 cm pieces with a kitchen knife and 4 g of each was placed in a separate SPME vial. The samples were pre-incubated for 5 min at 60 °C, then the fiber was exposed for 30 min at the same temperature to adsorb volatiles. The volatile compounds isolated by SPME were desorbed in the injector port on the GC×GC–ToF-MS system (Pegasus 4D LECO, St. Joseph, MI, USA). The GC was equipped with a DB-5 column (25 m × 0.2 mm × 0.33 μm, Agilent Technologies, Santa Clara, CA, USA) and a Supelcowax 10 (1.2 m × 0.1 mm × 0.1 μm, Supelco Bellefonte, PA, USA) as the second column. The injector temperature was set at 250 °C and injection was performed in a splitless mode. The gas flow was set at 0.8 mL/min. The primary oven temperature was programed as follows: 40 °C for 1 min, then rising at 6 °C/min to 200 °C, where it then was reduced at 25 °C/min to 235 °C, where it was held for 5 min. In the secondary oven, the following programming was used: Held at 65 °C for 1 min, then rising at 6 °C/min to 225 °C, where it then decreased at 25 °C/min to 260 °C, where it was held for 5 min. The transfer line temperature was 260 °C. The modulation time was 4 s. The time-of-flight mass spectrometer was operating at a mass range of *m/z* 38–388 and detector voltage −1700 V at 150 spectra/s. Total analysis time was 34.07 min. All analyses for any particular cultivar and preparation method were done in triplicates.

### 3.4. Data Analysis

Data were collected using the LECO ChromaTOF v.4.44 software (St. Joseph, MI, USA). Tentative identification was accomplished using the National Institute of Standards and Technology (NIST) library (version 2.0) of mass spectra. The calculations and basic statistical analysis were performed using Excel 2010 and Microsoft Office 2010. The principal component analysis (PCA) was performed by the SIMCA software, v. 14.1.0.2047 (MKS Unimetrix AB, Umea, Sweden).

## 4. Conclusions

The HS-SPME-GC×GC method was proposed for the identification of a large spectrum of volatiles from selected *Brassica* vegetables. This fast and efficient technique allowed for the determination of volatile compounds in different cultivars of broccoli, cauliflower, kohlrabi and Brussels sprouts. SPME is a method that provides a relatively fast volatile profiling of numerous samples. Despite many advantages of this technique, there are some issues which need to be mentioned here. First of all, there is no possibility to store volatile extracts (isolates), thus we face the problem of vegetable storage to analyze large sets of fresh raw vegetables. As presented, the freezing process caused some changes in the volatiles profile. Moreover, this is a non-exhaustive extraction method, so components with a low partition coefficient might be undetected, even if they are present in the sample in a relatively high concentration. Additionally, non-stable, easily reactive compounds can disintegrate, mainly due to high temperatures during thermal desorption from the fiber. The technique is very good for comparative purposes, pertinent for quantitative analysis; however, in the case of an external standard method used for quantitation, the impact of the matrix cannot be ignored, or alternatively, isotopomers of analyzed compounds could be used. Components from the following chemical groups were identified in those plants: Alcohols, aldehydes, isothiocyanates, nitriles, sulfides and others (unspecified). The proportions between the different groups were dependent on the vegetable type/species. The volatiles analyzed in raw and cooked vegetables showed changes occurring in the volatile fraction in cooked vegetables in comparison with raw ones. One of the most important differences in the volatile compounds concerned was a marked decrease in the contents of aldehydes and alcohols. The total amounts of sulfides decreased after cooking in all vegetables, except for broccoli. Isothiocyanate concentrations unexpectedly increased in most analyzed vegetables, while in the case of nitriles, a decrement was observed. Because all vegetables were frozen before analysis, the influence of freezing on the volatile fraction composition was also investigated. It was found that postharvest freezing of the *Brassica* vegetables induces biochemical changes that give rise to a significant modification of the compounds responsible for the aroma properties of those plants. Most of the changes are closely related to the activity of the LOX pathway enzymes. They are characterized by a high reduction in alcohol contents and an increase in aldehyde contents. Moreover, important changes were noted in the concentrations of bioactive components, e.g., isothiocyanates. The comparison of volatiles between fresh and thawed raw and cooked Brussels sprouts showed that after freezing, it is suggested to cook vegetables before consumption to maximize their beneficial properties.

## Supplementary Materials

The following are available online at http://www.mdpi.com/1420-3049/24/3/391/s1, Table S1. Main volatile compounds identified using GC×GC in raw and cooked broccoli varieties; Table S2. Main volatile compounds identified using GC×GC in raw and cooked cauliflower varieties; Table S3. Main volatile compounds identified using GC×GC in raw and cooked kohlrabi varieties; Table S4. Main volatile compounds identified using GC×GC in raw and cooked Brussels sprouts varieties; Table S5. Compounds and their corresponding numbers used in PCA analysis (Figure 1).
