# Supplementary material for: Volatile Compounds of Selected Raw and Cooked Brassica Vegetables
Source: Molecules. 2019 Jan 22;24(3):391. doi: 10.3390/molecules24030391 (PMC6385023; doi:10.3390/molecules24030391)
Supplement: Supplementary file 1 [file molecules-24-00391-s001.pdf]

Table S1. Main volatile compounds identified using GC × GC in raw and cooked broccoli varieties.

Compound	IR	Peak area (10^5)					
		Malibu raw	Malibu cooked	Covina raw	Covina cooked	'2970'' raw	"2970" cooked
**Aldehydes**							
Propanal	517	- ± -	35,88 ± 1,3	74,05 ± 8	33,9 ± 20,69	208,78 ± 24,89	26,45 ± 3,31
Butanal	560	- ± -	9,9 ± 1,3	26,76 ± 2,68	9 ± 2,02	19,34 ± 0,67	- ± -
2-butenal	608	- ± -	85,4 ± 9,57	296,6 ± 2,64	112,29 ± 34,84	312,53 ± 46,52	35,41 ± 18,45
2-Pentenal, (E)-	752	80,5 ± 3,86	63,16 ± 8,31	359,1 ± 16,6	73,42 ± 23,21	197,91 ± 61,73	78,16 ± 7,72
3-Hexenal, (Z)-	795	157 ± 14,81	0 ± 0	121,9 ± 1,55	- ± -	61,05 ± 9,97	- ± -
Hexanal	797	772 ± 66,61	216,8 ± 44,81	693,4 ± 94,9	270,05 ± 48,25	634,76 ± 40,78	297,34 ± 43,28
2-Hexenal	852	471 ± 52,3	61,95 ± 20,11	787,5 ± 65,2	100,1 ± 16,53	1471,41 ± 736	171,35 ± 36,82
2,4-Hexadienal, (E,E)-	898	83,9 ± 13,19	- ± -	88,97 ± 15,7	- ± -	43,14 ± 23,28	2,51 ± 1,84
Heptanal	900	18,3 ± 2,99	29,93 ± 5,74	50,28 ± 2,56	52,04 ± 7,54	43,19 ± 4,99	34,01 ± 6,72
2-heptanal	959	- ± -	72,84 ± 12,36	26,6 ± 0,31	110,38 ± 50,71	26,24 ± 1,5	37,69 ± 25,31
Benzaldehyde	972	73,9 ± 25,21	17,38 ± 2,49	329,3 ± 20,3	34,11 ± 5,92	182,26 ± 37,4	20,41 ± 5,72
2,4-Heptadienal, (E,E)-	993	286 ± 87,17	35,8 ± 7,36	506,6 ± 32,9	60,31 ± 24,71	357,96 ± 81,01	30,98 ± 14,58
Octanal	1008	4,89 ± 0,08	- ± -	23,61 ± 1,47	- ± -	12,27 ± 1,16	- ± -
Benzeneacetaldehyde	1055	172 ± 20,78	16,28 ± 6,17	255,9 ± 7,63	20,57 ± 1,51	173,95 ± 8,98	21,95 ± 7,43
2-Octenal, (E)-	1064	- ± -	44,11 ± 11,1	69,74 ± 6	37,15 ± 17,22	33,64 ± 0,5	24,83 ± 10,01
Nonanal	1108	29,9 ± 0,97	110,96 ± 47,88	90,86 ± 4,84	172,55 ± 21,09	55,46 ± 4,21	114,46 ± 14,5
**Total**	**-**	**2149,23**	**800,37**	**3801,14**	**1085,88**	**3833,89**	**895,54**
**Isothiocyanates**							
Allyl isothiocyanate	871	- ± -	- ± -	±	- ± -	- ± -	1,14 ± 0,11
Butane, 2-isothiocyanato-	932	- ± -	- ± -	±	- ± -	- ± -	0,77 ± 0,07
Isobutyl isothiocyanate	957	2,8 ± 0,02	- ± -	- ± -	- ± -	1,78 ± 0,3	1,71 ± 0,13
Hexane, 1-isocyanato-	971	0,47 ± 0,01	- ± -	0,56 ± 0,05	- ± -	0,38 ± 0,12	0,63 ± 0,02
1-Butene, 4-isothiocyanato-	998	- ± -	8,29 ± 0,66	- ± -	2,48 ± 1,06	- ± -	3,77 ± 0,13
Cyclopentyl isothiocyanate	1003	2,9 ± 1,02	- ± -	0,61 ± 0,25	- ± -	1,36 ± 1,63	- ± -
Butane, 1-isothiocyanato-	1004	- ± -	2,26 ± 0,67	- ± -	3,84 ± 1,13	- ± -	3,54 ± 0,1
2-Methylbutyl isothiocyanate	1066	- ± -	12,07 ± 3,37	- ± -	- ± -	- ± -	2,66 ± 0,22
Benzene, (2-isothiocyanatoethyl)-	1461	- ± -	5,18 ± 1,29	- ± -	0,36 ± 0,18	- ± -	0,7 ± 0,46
Propane, 1-isothiocyanato-	1484	17,6 ± 0,74	2,42 ± 0,85	- ± -	0,18 ± 0,03	5,26 ± 0,94	4,66 ± 0,26
**Total**	**-**	**23,77**	**30,22**	**1,14**	**6,86**	**8,78**	**22,23**
**Other sulfur compounds**							
Sulfides (di, tri, tetra, penta)	-	349 ± 58,82	794,36 ± 157,96	366,1 ± 81,5	1030,59 ± 380,9	593,39 ± 175,4	1497,75 ± 113,25
Thiocyanic acid, methyl ester	709	316 ± 45,88	6,84 ± 1,14	536,1 ± 45,5	9,84 ± 1,1	577,47 ± 71,55	19,28 ± 5,83
Thiophene, 2-methyl-	771	1,17 ± 0,16	0,91 ± 0,42	3,13 ± 0,21	2,08 ± 1,17	3,32 ± 1,45	3,16 ± 0,04
Thiophene, 2-ethyl-	869	106 ± 15,69	29,8 ± 15,26	60,17 ± 1,44	60,31 ± 23,17	76,89 ± 7,53	- ± -
Thiophene, 2-pentyl-	1170	0,57 ± 0,07	- ± -	0,3 ± 0,02	- ± -	0,53 ± 0,46	±
**Total**	**-**	**772,22**	**831,91**	**956,86**	**1102,83**	**1251,60**	**1520,19**
**Nitriles**							
Butanenitrile, 3-methyl-	728	- ± -	- ± -	23,3 ± 28	- ± -	- ± -	- ± -
Pentanenitrile, 4-methyl-	867	10,4 ± 2,61	- ± -	- ± -	- ± -	- ± -	- ± -
Hexanenitrile	876	11 ± 4,31	- ± -	22,61 ± 1,32	- ± -	21,75 ± 13,46	6,7 ± 0,17
Hexanenitrile, 5-methyl-	945	14,6 ± 3,38	- ± -	5,63 ± 0,32	- ± -	25,3 ± 14,46	10,72 ± 0,79

Heptanenitrile	981	12,5 ± 2,14	4,09 ± 0,94	58,59 ± 4,46	2,42 ± 3,2	50,26 ± 16,77	12,1 ± 4,05
Benzyl nitrile	1150	1,22 ± 0,23	- ± -	- ± -	- ± -	- ± -	- ± -
Octanenitrile	1185	- ± -	- ± -	1,32 ± 1,12	- ± -	- ± -	0 ± 0
Pentanenitrile, 5-(methylthio)-	1209	- ± -	7,7 ± 9,67	3,12 ± 0,37	11,19 ± 6,44	2,28 ± 1,57	31,96 ± 6,16
Benzenepropanenitrile	1250	47 ± 11,72	- ± -	79,59 ± 4,27	- ± -	28,05 ± 16,51	- ± -
**Total**	-	**96,72**	**11,78**	**194,16**	**13,61**	**127,64**	**61,47**
**Alcohols**							
1-penten-3-ol	664	487 ± 12,15	27,2 ± 6,63	866,4 ± 207	24,53 ± 4,17	472,77 ± 0	28,8 ± 6,46
1-pentanol	763	5,37 ± 1,24	297,69 ± 48,9	101,3 ± 26,1	235,85 ± 36,82	14,26 ± 0,5	170,2 ± 9,83
2-penten-1-ol	765	216 ± 67,95	- ± -	457,1 ± 123	- ± -	232,84 ± 63,22	- ± -
2-hexen-1-ol	864	11,3 ± 3,34	- ± -	151,5 ± 91	- ± -	96,18 ± 27,05	- ± -
1-hexenol	867	129 ± 7,58	- ± -	417 ± 102	- ± -	520,61 ± 163,8	- ± -
1-octen-3-ol	986	27,6 ± 3,89	- ± -	- ± -	- ± -	68,98 ± 32,27	- ± -
**Total**	-	**876,77**	**324,89**	**1993,25**	**230,38**	**1405,64**	**199,00**
**Other compounds**							
1-penten-3-one	670	563 ± 49,01	101,6 ± 10,76	411,6 ± 185	112,13 ± 27,11	266,97 ± 2,65	112,54 ± 28,39
Furan, 2-ethyl	695	983 ± 146,2	- ± -	806 ± 18,5	- ± -	1214,99 ± 310,2	796,79 ± 51,94
furan, 2-pentyl	996	47,5 ± 9,73	0,62 ± 0,28	182,2 ± 3,05	0,62 ± 0,22	145,14 ± 19,89	358,1 ± 61,91
3,5-octadien-2-one	1073	32,3 ± 18,19	- ± -	277 ± 221	- ± -	87,83 ± 96,66	28,28 ± 3,03
**Total**	-	**1625,54**	**102,22**	**1676,69**	**112,75**	**1714,93**	**1295,70**

Table S2. Main volatile compounds identified using GC × GC in raw and cooked cauliflower varieties.

**Compound**	**IR**	**Peak area (10^5)**					
		**Oviedo raw**	**Oviedo cooked**	**Liria raw**	**Liria cooked**	**Charlotte raw**	**Charlotte cooked**
**Aldehydes**							
2-Propenal	514	55,68 ± 7,36	6,39 ± 0,5	70,03 ± 42,77	6,71 ± 2,66	52,24 ± 17,39	31,62 ± 7,06
Propanal	517	140,9 ± 39,27	- ± -	- ± -	- ± -	394,8 ± 27,62	- ± -
Butanal	561	38,95 ± 3,66	5,68 ± 1,97	79,34 ± 16,24	9,69 ± 3,62	38,01 ± 17,34	25,6 ± 7,4
2-Butenal	607	141 ± 17,32	33,05 ± 15,32	236,2 ± 47,68	179,69 ± 39,89	550,2 ± 585,65	71,45 ± 17,04
Butanal, 3-methyl-	619	46,89 ± 3,42	2,58 ± 0,83	38,08 ± 7,28	15,28 ± 2,22	70,93 ± 51,04	18,07 ± 0,29
Butanal, 2-methyl-	631	- ± -	8,92 ± 1,84	- ± -	38,53 ± 5,73	- ± -	63,57 ± 10,89
Pentanal	670	- ± -	355,96 ± 78,55	- ± -	331,94 ± 57,81	- ± -	136,9 ± 86,68
2-Pentenal, (E)-	752	322,4 ± 9,09	45,13 ± 2,93	300,9 ± 63,77	88,69 ± 8,04	239,8 ± 5,57	173,3 ± 19,79
3-Hexenal, (Z)-	759	108,8 ± 1,18	- ± -	104 ± 42,6	0 ± 0	48,3 ± 8,48	0 ± 0
Heksanal	797	19,67 ± 2,25	415,63 ± 83	314,2 ± 155,2	101,45 ± 13,54	794,2 ± 193,56	779,5 ± 115,16
2-Hexenal	852	754,6 ± 56,39	53,88 ± 17,01	1106 ± 163,2	120,31 ± 17,93	236,3 ± 92,4	90,95 ± 21,91
2,4-Hexadienal, (E,E)-	898	59,94 ± 8,66	2,21 ± 0,72	163,3 ± 51,34	6,47 ± 1,07	194,2 ± 25,05	12,06 ± 1,18
Heptanal	901	163,4 ± 3,91	41,87 ± 4,29	156,9 ± 45,44	80,34 ± 8,31	199,9 ± 25,9	133,3 ± 47,54
2-Heptenal, (Z)-	959	58,54 ± 7,54	20,72 ± 13,35	66,83 ± 44,06	31,25 ± 7,33	159,6 ± 59,69	42,46 ± 8,96
Benzaldehyde	972	284,8 ± 45,05	15,41 ± 0,88	410,5 ± 0,31	30,65 ± 1,5	703,6 ± 549,32	56 ± 8,04
2,4-Heptadienal, (E,E)-	996	598,8 ± 82,1	42,51 ± 12,52	871,6 ± 93,13	127,71 ± 58,68	711,2 ± 66,2	21,05 ± 4,62
Octanal	1008	98,34 ± 6,64	62,57 ± 16,37	93,86 ± 2,47	98,45 ± 29,78	100,3 ± 47,64	123,2 ± 11,26

Benzeneacetaldehyde	1055	80,9	±	2,48	5,12	±	1,45	109,6	±	69,73	7,69	±	1,34	146,3	±	12,19	20,89	±	2,72
2-Octenal, (E)-	1064	151,9	±	42,37	15,53	±	3,92	116,8	±	18,22	36,59	±	5,17	330,8	±	104,34	44,34	±	12,82
Nonanal	1108	162,2	±	16,56	219,33	±	24,29	98,45	±	44,36	201,66	±	83,62	57,09	±	16,32	132,3	±	71,74
2,4-Octadienal, (E,E)-	1119	5,36	±	2,53	-	±	-	16,59	±	8,22	-	±	-	14,68	±	0,15	-	±	-
2-Nonenal, (E)-	1164	19,43	±	3,83	-	±	-	42,61	±	26,05	11,25	±	3,93	66,71	±	36,23	50,58	±	33,48
Decanal	1209	24,16	±	1,78	10,61	±	0,36	26,77	±	2,97	20,18	±	7,79	66,68	±	30,07	20,89	±	10,13
2,4-Nonadienal, (E,E)-	1222	21,45	±	8,1	-	±	-	39,28	±	4,9	-	±	-	84	±	4,34	71,45	±	17,04
2,4-Decadienal, (E,E)-	1287	-	±	-	8,55	±	4,3	-	±	-	41,06	±	7,79	-	±	-	19,16	±	14,31
**Total**	-		**3357,94**			**1371,64**			**4461,94**			**1585,60**			**5259,77**			**2123,63**	
**Isothiocyanates**						±						±						±	
allyl isothiocyanate	871	79,45	±	1,56	33,23	±	8,03	205,8	±	50,63	315,98	±	39,64	212,3	±	52,46	175,9	±	16,92
Isobutyl isothiocyanate	957	2,84	±	0,41	2,46	±	0,78	102	±	62,49	33,32	±	4,03	6,66	±	1,49	43,84	±	1,21
Cyclopropane, isothiocyanato-	966	-	±	-	-	±		7,98	±	4,62	8,49	±	1,45	-	±	-	13,03	±	2,25
1-Butene, 4-isothiocyanato-	998	-	±	-	2,66	±	0,61	-	±	-	23,12	±	3,54	-	±	-	168,7	±	89,06
Cyclopentyl isothiocyanate	1003	-	±	-	-	±		17,98	±	8,05	0	±	0	9,25	±	4,58	0	±	0
2-Methylbutyl isothiocyanate	1066	6,09	±	0,01	4,67	±	1,55	12,91	±	2,68	47,99	±	2,1	9,53	±	1,09	51,62	±	5,59
Propane, 1-isocyanato-2-methyl-	1348	1,19	±	0,05		±		-	±	-	0	±	0	-	±	-	0	±	0
Propane, 1-isothiocyanato-3-(methylthio)-	1467	1,57	±	0,94	2,22	±	0,85	1,71	±	0,76	44,35	±	11,4	0,91	±	0,37	16,03	±	1,49
Propane, 1-isothiocyanato-	1514	-		-	1,36	±	0,65	-	±	-	7,12	±	0,71	-	±	-	15,94	±	5,87
**Total**	-		**91,14**			**46,6**			**348,31**			**480,37**			**238,65**			**485,05**	
**Other sulfides**																			
Sulfides (di, tri, tetra, penta)	-	694,6	±	81,23	433,82	±	27,63	1022	±	134,5	841,46	±	164,14	748,2	±	394,87	1342	±	115,01
Thiocyanic acid, methyl ester	709	419,3	±	12,53	4,56	±	0,31	565,8	±	43,09	20,12	±	3,08	333,7	±	28,15	48,15	±	2,56
Thiophene, 2-ethyl-	869	136,3	±	29,36	-	±	-	108,3	±	18,56	-	±	-	188	±	27,46	-	±	-
Thiocyanic acid, 2-propenyl ester	873	3,34	±	2,39	5,16	±	1,47	4,42	±	1,1	55,04	±	7,84	-	±	-	50,24	±	20,4
S-Methyl pentanethioate	998	1,88	±	0,3	-	±	-	8,19	±	1,48	-	±	-	3,58	±	1,29	-	±	-
S-Methyl methanethiosulphonate	1072	4,52	±	3,33	-	±	-	4,9	±	0,66	-	±	-	-	±	-	-	±	-
**Total**	-		**1259,95**			**443,55**			**1713,74**			**916,61**			**1273,52**			**1440,71**	
**Nitriles**																			
3-Butenenitrile	621	660,2	±	75,91	-	±	-	685,2	±	186,4	0	±	0	-	±	-	0	±	0
2-butanenitrile	622	-	±	-	4,16	±	0,78	-	±	-	20,51	±	2,9	710,7	±	80,89	70,86	±	10,81
Butanenitrile, 3-methyl-	728	51,37	±	15,12	1,6	±	0,24	159,6	±	94,43	-	±	-	113,3	±	99,12	20,39	±	5,99
Pentanenitrile, 4-methyl-	840	31,09	±	0,47	-	±	-	67,55	±	18,31	-	±	-	77,73	±	34,66	0	±	0
Hexanenitrile	876	17,71	±	2,13	-	±	-	26,11	±	1,59	-	±	-	25,49	±	7,64	0	±	0
Hexanenitrile, 5-methyl-	945	2,25	±	0,84	-	±	-	13,8	±	3,17	-	±	-	-	±	-	0	±	0
Heptanonitrile	981	8,28	±	0,98	-	±	-	-	±	-	-	±	-	-	±	-	0	±	0
Butanenitrile, 4-(methylthio)-	1092	39,34	±	2,07	27,95	±	9,74	11,37	±	5,09	121,8	±	50,43	141,9	±	192,45	205	±	27,44
Benzyl nitrile	1150	3,23	±	0,16	-	±	-	3,59	±	0,23	0	±	0	-	±	-	0	±	0
Pentanenitrile, 5-(methylthio)-	1209					±					2,67	±	1,25				19,65	±	1,22
Benzenepropanenitrile	1250	15,95	±	0,12	-	±	-	8,37	±	2,25	0	±	0	26,03	±	8,41	0	±	0
**Total**	-		**829,41**			**33,72**			**975,6**			**144,97**			**1095,14**			**315,89**	
**Alcohols**																			
1-Penten-3-ol	664	633,9	±	35,74	15,14	±	1,85	319,9	±	248,4	23,06	±	6,51	361,2	±	273,02	196,9	±	43,08
1-Pentanol	763					±					96,19	±	14,66				954,3	±	9,06
2-Penten-1-ol, (Z)-	765	160,7	±	84,83		±		397,7	±	17,42	0	±	0	284	±	274,67	18,64	±	4,23
1-Hexanol	867	369,1	±	24,18		±		128,2	±	17,31	0	±	0	480	±	326,76		±	

1-Octen-3-ol	986	228,7 ± 3,12	18,95 ± 14,87	309,4 ± 121,2	17,54 ± 3,23	215,6 ± 253,52	170,2 ± 41,5
**Total**	-	**1392,47**	**34,09**	**1155,21**	**136,76**	**1340,75**	**1340,01**
**Other components**			±		0 ± 0		0 ± 0
Furan, 2-ethyl-	695	947,7 ± 144,5	434,91 ± 75,31	1071 ± 581,2	823,65 ± 132,95	622,3 ± 374,47	1065 ± 79,74
3,5-Octadien-2-one, (E,E)-	1073	272,5 ± 64,44	7,94 ± 1,59	369,3 ± 162,6	22,01 ± 7,42	375,6 ± 403,15	21,03 ± 1,27
**Total**	-	**1220,2**	**442,8**	**1439,85**	**845,65**	**997,87**	**1084,68**

Table S3. Main volatile compounds identified using GC × GC in raw and cooked kohlrabi varieties.

**Compound**	**IR**	**Peak area (10^5)**							
		**Konmar raw**	**Konmar cooked**	**Kolibri raw**	**Kolibri cooked**	**Konan raw**	**Konan cooked**	**Kordial raw**	**Kordial cooked**
**Aldehydes**									
2-Butenal	608	12,9 ± 1,73	27,5 ± 26,9	105,4 ± 5,67	3,03 ± 2,45	6,04 ± 0,66	5,37 ± 12,6	178 ± 104,35	68,2 ± 49,1
Hexanal	795	3,2 ± 0,32	248 ± 44,5	1,33 ± 0,22	102 ± 60,6	2,59 ± 0,56	42,7 ± 182	80,5 ± 13,37	118 ± 85
Benzaldehyde	972	4,95 ± 0,23	17,2 ± 2,27	3,87 ± 0,11	4,76 ± 1,34	4,51 ± 0,09	2,87 ± 7,68	27,9 ± 35,69	22,2 ± 6,61
Octanal	1008	- ± -	207 ± 19,7	- ± -	27,2 ± 6,74	1,63 ± 0,17	14,4 ± 37,5	7,44 ± 1,94	28 ± 5,73
Benzacetaldehyde	1055	0,64 ± 0,38	5,2 ± 1,7	3,76 ± 0,29	1,39 ± 0,19	1,16 ± 0,01	0,76 ± 1,27	45,7 ± 22,05	2,21 ± 1,04
Nonanal	1108	18,4 ± 3,04	326 ± 70,5	8,76 ± 0,75	83 ± 6,1	20,8 ± 0,29	30,8 ± 60,2	65,5 ± 6,1	58,6 ± 17,2
**Total**	-	40,13	830,88	123,11	221,39	36,69	96,94	405,23	297,23
**Isothiocyanates**									
1-Butene, 4-isothiocyanato-	998	21,1 ± 0,92	214 ± 71,2	47,76 ± 0,53	1,48 ± 0,09	15,8 ± 4,75	14,1 ± 9,55	15,4 ± 1,07	31,7 ± 14,2
Methane, isothiocyanato-	749	5,2 ± 0,89	- ± -	13,1 ± 0,6	- ± -	4,65 ± 0,37	- ± -	- ± -	- ± -
Isopropyl isothiocyanate	832	- ± -	- ± -	- ± -	- ± -	- ± -	- ± -	1,81 ± 0,18	- ± -
Allyl isothiocyanate	871	- ± -	- ± -	5,56 ± 0,04	0,24 ± 0,1	2,39 ± 0,19	3,55 ± 5,18	55,2 ± 65,2	34,5 ± 6,42
Butane, 2-isothiocyanato-	932	- ± -	0,97 ± 0,8	4,67 ± 0,53	0,36 ± 0,26	0,27 ± 0	- ± -	- ± -	0,47 ± 0,24
Isobutyl isothiocyanate	957	- ± -	4,2 ± 2,26	- ± -	0,33 ± 0,05	3,39 ± 0,01	- ± -	26,1 ± 34,79	- ± -
Butane, 1-isothiocyanato-	1004	- ± -	- ± -	6,95 ± 0,61	- ± -	0,54 ± 0,03	- ± -	- ± -	- ± -
2-Methylbutyl isothiocyanate	1066	5,16 ± 1,82	11,1 ± 5,93	98,82 ± 11	1,69 ± 1,02	9,2 ± 0,23	0,12 ± 0,03	9,84 ± 12,45	12,8 ± 11
Butane, 1-isothiocyanato-3-methyl-	1066	- ± -	- ± -	1,99 ± 0,32	- ± -	0 ± 0	- ± -	- ± -	- ± -
n-Pentyl isothiocyanate	1103	1,42 ± 0,27	359 ± 118	19,23 ± 3,27	- ± -	- ± -	0,03 ± 0	- ± -	229 ± 102
4-Methylpentyl isothiocyanate	1167	- ± -	- ± -	16,36 ± 1,78	- ± -	1,76 ± 0,09	0 ± 0	- ± -	- ± -
Hexane, 1-isothiocyanato-	1207	21,5 ± 0,38	6,5 ± 7,26	37,7 ± 6,32	- ± -	11,6 ± 0,42	- ± -	19,8 ± 13,51	1,61 ± 2,03
Heptane, 1-isothiocyanato-	1269	1,47 ± 0,02	- ± -	2,5 ± 0,57	- ± -	0,74 ± 0,01	- ± -	0,66 ± 0,4	±
Benzene, (isothiocyanatomethyl)-	1461	1,28 ± 0,01	1,04 ± 0,35	26,38 ± 0,05	- ± -	3,24 ± 0,56	2,65 ± 1,91	1,62 ± 1,6	12,2 ± 13,6
Propane, 1-isothiocyanato-3-(methylthio)-	1467	365 ± 8,09	68,5 ± 38,9	299 ± 81,8	3,64 ± 1,37	276 ± 67,4	- ± -	23,9 ± 12,03	89,1 ± 116
Propane, 1-isothiocyanato-	1484	- ± -	- ± -	- ± -	- ± -	- ± -	- ± -	- ± -	- ± -
Octane, 1-isothiocyanato-	1514	1,12 ± 0,04	- ± -	1,05 ± 0,07	- ± -	0,46 ± 0	- ± -	1,29 ± 0,71	- ± -
1-Butene, 4-isothiocyanato-1-(methylthio)-	1534	1,37 ± 0,1	- ± -	3,53 ± 0,01	- ± -	1,05 ± 0,19	2,49 ± 1,44	1,15 ± 0,5	±
Benzene, (2-isothiocyanatoethyl)-	1588	277 ± 12	- ± -	764,3 ± 48,5	7,94 ± 1,3	147 ± 30,4	0 ± 0	194 ± 72,79	- ± -

**Total**	-	**701,6**	**664,95**	**1348,82**	**15,68**	**478,17**	**22,97**	**350,3**	**410,73**
**Other sulfides**									
Sulfides (di, tri, tetra, penta)	-	9305 ± 2203	3488 ± 1602	3189 ± 118	2510 ± 560	8890 ± 148	1982 ± 398	7004 ± 1145,4	5428 ± 995
Thiocyanic acid, methyl ester	709	139 ± 8,54	20,4 ± 8,2	259,4 ± 9,45	- ± -	127 ± 20,6	1,15 ± 1,06	418 ± 83,92	18 ± 13,4
**Total**	-	**9444,06**	**3508,44**	**3448,34**	**2510,28**	**9017,13**	**1982,76**	**7421,75**	**5445,53**
**Nitriles**					±		0 ± 0		±
3-Butenenitrile	621	- ± -	2,54 ± 0,25	- ± -	- ± -	- ± -	0 ± 0	26,6 ± 22,78	5,18 ± 1,7
2-Pentenenitrile	760	- ± -	±	0 ± 0	- ± -	- ± -	0 ± 0	- ± -	±
Pentanenitrile, 4-methyl-	840	1,65 ± 0,77	8,61 ± 1,81	5,54 ± 0,36	5,62 ± 0,73	1,74 ± 0,27	1,66 ± 0,21	- ± -	13,2 ± 4,72
Hexanenitrile	876	1,63 ± 0,55	4,32 ± 0,82	5,72 ± 0,15	2,43 ± 0,62	2,49 ± 0,11	1,43 ± 0,38	- ± -	7,57 ± 4,02
Butanenitrile, 4-(methylthio)-	1092	244 ± 21,4	280 ± 158	601,6 ± 80,6	- ± -	349 ± 0,56	34 ± 8,3	1244 ± 44,95	275 ± 124
Benzyl nitrile	1150	2,43 ± 0,05	1,53 ± 0,19	3,74 ± 0,46	- ± -	4,12 ± 1,01	0 ± 0	13 ± 5,03	31,6 ± 45,3
Octanenitrile	1185	- ± -	0,48 ± 0,11	1,63 ± 0,39	- ± -	0,99 ± 0,1	0 ± 0	- ± -	6,29 ± 12,1
Pentanenitrile, 5-(methylthio)-	1209	955 ± 141	516 ± 113	1463 ± 4,35	- ± -	1588 ± 187	111 ± 26,9	- ± -	342 ± 295
Benzenepropanenitrile	1250	27,4 ± 1,54	32,9 ± 5,76	116,4 ± 1,45	12,5 ± 8,36	28,6 ± 1,15	0 ± 0	31,1 ± 16,03	14,3 ± 6,89
**Total**	-	**1232,55**	**846,16**	**2192,31**	**20,50**	**1975,32**	**147,82**	**1315,07**	**685,67**

Table S4. Main volatile compounds identified using GC × GC in raw and cooked Brussel sprouts varieties.

**Compound**	**IR**	**Peak area (105)**									
		Maximus raw	Maximus cooked	Marte raw	Marte cooked	Ajax raw	Ajax cooked	Neptuno raw	Neptuno cooked	Profitus raw	Profitus cooked
**Aldehydes**											
Propanal	517	200,96 ± 31,46	304,05 ± 152	164,5 ± 17,98	17,95 ± 8,83	154,07 ± 50,81	0 ± 0	118,94 ± 7,03	0 ± 0	212,2 ± 14,5	0 ± 0
Butanal	560	17,51 ± 1,22	0 ± 0	17,55 ± 2,13	0 ± 0	15,27 ± 11,32	0 ± 0	10,27 ± 1	0 ± 0	34,63 ± 2,54	0 ± 0
2-Butenal	608	730,64 ± 52,34	266,36 ± 19,7	337,5 ± 178,3	57,37 ± 11,3	161,41 ± 10,24	13,03 ± 5,48	228,63 ± 29,8	57,37 ± 11,3	1176 ± 78,1	475,2 ± 162,3
Butanal, 2-methyl-	632	86,97 ± 1,48	97,12 ± 24,3	129,7 ± 22,25	18,17 ± 4,81	70,34 ± 33,35	6,84 ± 0,95	71,07 ± 5,61	0 ± 0	94,7 ± 0,65	18,8 ± 1,13
Pentanal	681	0 ± 0	337,28 ± 162	0 ± 0	295,47 ± 47,3	0 ± 0	114,7 ± 36,6	0 ± 0	295,5 ± 47,3	0 ± 0	273,3 ± 13,98
2-Pentenal, (E)-	752	213,32 ± 8,03	58,56 ± 50,5	208 ± 37,85	3,82 ± 0,5	150,76 ± 152,9	2,81 ± 0,79	105,45 ± 14,1	0 ± 0	102,8 ± 18,2	9,21 ± 0,77
3-Hexenal, (Z)-	795	81,95 ± 4,47	2,07 ± 1,27	70,87 ± 3,08	0,97 ± 0,59	55,09 ± 12,26	0,56 ± 0,21	70,18 ± 6,19	0,62 ± 0,33	63,82 ± 4,3	0,64 ± 0,07
Hexanal	797	145,62 ± 37,26	860,01 ± 300	271,8 ± 24,67	147,91 ± 26,2	173,25 ± 99,59	100,2 ± 16,6	434,1 ± 22,6	147,9 ± 26,2	383 ± 16,7	165,8 ± 8,3
2-Hexenal	852	481,2 ± 29,33	169,72 ± 16,1	230,9 ± 22,1	23,8 ± 8,18	167,05 ± 65,56	21,53 ± 6,62	205,71 ± 17,9	23,8 ± 8,18	269,6 ± 1,37	38,81 ± 7,63
2-Hexenal, (E)-	852	123,53 ± 5,19	169,72 ± 16,1	51,34 ± 3,77	17,12 ± 5,5	37,07 ± 13,84	0,56 ± 0,21	68,73 ± 14,6	21,87 ± 6,68	67,45 ± 0,57	38,81 ± 7,63
2,4-Hexadienal, (E,E)-	898	189,4 ± 3,24	25,51 ± 2,26	107,1 ± 12,19	0 ± 0	122,54 ± 47,25	0 ± 0	160,39 ± 86,1	0 ± 0	174,1 ± 58,3	22,01 ± 8,19
2-Heptenal, (Z)-	959	85,15 ± 9,34	142,53 ± 71,4	38,49 ± 5,02	36,33 ± 7,89	71,32 ± 29,51	0 ± 0	69,75 ± 0,06	36,33 ± 7,89	70,19 ± 0,32	56,88 ± 15,85
Benzaldehyde	972	1384,5 ± 95,9	350,62 ± 88,1	1787 ± 191,6	139,62 ± 15,5	1918,9 ± 599,1	86,91 ± 15,1	1328,2 ± 101	139,6 ± 15,5	257,6 ± 54,3	80,73 ± 11,74
2,4-Heptadienal, (E,E)-	993	212,79 ± 6,02	481,66 ± 50,5	130 ± 10,45	246,38 ± 162	216,69 ± 40,87	70,95 ± 23,5	249,37 ± 111	246,4 ± 162	139,3 ± 22,2	165,8 ± 8,3
Benzeneacetaldehyde	1055	112,16 ± 18,31	36,52 ± 8,61	95,48 ± 12,54	28,61 ± 3,16	130,11 ± 6,26	8,66 ± 2,91	97,93 ± 2,47	34,63 ± 12,2	58,44 ± 0,87	17,9 ± 2,54
Nonanal	1108	61,78 ± 2,67	107,36 ± 16,2	103,9 ± 18,11	107,92 ± 16,5	98,61 ± 20,69	24,42 ± 7,45	113,28 ± 97,5	107,9 ± 16,5	65,29 ± 8,36	71,23 ± 17,16
2,6-Nonadienal, (E,E)-	1158	51,25 ± 5,23	10,88 ± 1,76	51,66 ± 1,59	16,5 ± 4,06	48,76 ± 9,29	5,88 ± 1,56	41,66 ± 3,38	12,54 ± 6,07	108,5 ± 11,5	9,26 ± 3,01
Decanal	1209	18,2 ± 5,01	28,46 ± 4,47	18,8 ± 3,7	19,26 ± 2,33	14,53 ± 1,41	4,57 ± 0,97	14,8 ± 1,42	19,39 ± 8,16	21,57 ± 7,13	12,59 ± 2,67
2-Propenal	1250	90,1 ± 3,23	129,33 ± 70,2	113,9 ± 12,24	64,61 ± 24,9	142,61 ± 20,54	0 ± 0	92,15 ± 1,36	64,61 ± 24,9	121,2 ± 31,9	69,79 ± 9,92
**Total**	-	**4286,99**	**3577,76**	**3928,10**	**1241,81**	**3748,32**	**461,68**	**3480,63**	**1208,47**	**3420,33**	**1526,75**
**Isothiocyanates**											
Cyclopropane, isothiocyanato-	866	0 0	702,1 ± 27,5	0 ± 0	407,64 ± 94	0 ± 0	233,5 ± 42,5	0 ± 0	407,6 ± 94	0 ± 0	337,5 ± 11,91
Allyl Isothiocyanate	871	2841,6 ± 58,4	7902,8 ± 602	2673 ± 1015	4959,1 ± 1471	2796 ± 284,2	2339 ± 264	2516,9 ± 420	4959 ± 868	2701 ± 178	1189 ± 226,5

Isopropyl isothiocyanate	932	2,69 ± 0,72	7,71 ± 2,21	28,09 ± 9,53	444,38 ± 18,8	25,39 ± 2,87	1,11 ± 0,58	10,88 ± 0,56	12,8 ± 2,78	18,43 ± 5,51	10,98 ± 1,77
Isobutyl isothiocyanate	957	157,73 ± 13,91	497,99 ± 22,5	329,6 ± 59,26	444,38 ± 18,8	383,35 ± 75,83	154,7 ± 29,3	406,22 ± 10,4	444,4 ± 18,8	165,6 ± 65,9	293,2 ± 129,4
Hexane, 1-isocyanato-	971	1,61 ± 0,05	0,58 ± 0,05	1,88 ± 0,14	0,86 ± 0,22	1,99 ± 1,23	0 ± 0	2,38 ± 0,78	0,63 ± 0,19	1,82 ± 0,31	0,58 ± 0,07
1-Butene, 4-isothiocyanato-	998	859,46 ± 383	105,11 ± 83,9	461,1 ± 28,94	104,68 ± 38,9	979,47 ± 53,29	18,47 ± 14,5	1258,8 ± 276	104,7 ± 38,9	444,2 ± 300	44,4 ± 26,39
2-Methylbutyl isothiocyanate	1066	81,7 ± 2,69	172,31 ± 41,5	141,2 ± 14,83	213,74 ± 7,4	105 ± 30,3	66,21 ± 15,1	177,93 ± 18,7	213,7 ± 7,4	28,27 ± 6,88	121,1 ± 31,69
Hexane, 1-isothiocyanato-	1269	2,28 ± 0,87	16,23 ± 12	19,4 ± 5,75	13,34 ± 3,78	46,12 ± 6,51	8,4 ± 2,33	9,93 ± 0,73	65,98 ± 5,84	27,28 ± 4,12	23,62 ± 3,02
Benzene, (isothiocyanatomethyl)-	1461	195,13 ± 65,86	570,11 ± 172	271,4 ± 77,77	33,84 ± 7,79	688,14 ± 192,2	274,3 ± 139	570,92 ± 18,9	33,84 ± 7,79	6,96 ± 0,98	8,9 ± 5,04
Benzene, (2-isothiocyanatoethyl)-	1588	2,64 ± 0,27	39,58 ± 15,6	29,69 ± 7,94	33,84 ± 7,79	380,1 ± 100,4	47,53 ± 1,37	347,32 ± 411	280,8 ± 29,9	83 ± 42,2	53,34 ± 18,49
**Total**	-	**4144,87**	**10014,57**	**3954,94**	**6655,80**	**5405,53**	**3143,10**	**5301,26**	**6523,55**	**3476,53**	**2082,78**
**Other sulfides**			±				±				
Sulfides (di, tri, tetra, penta)	-	476,61 ± 160,7	682,18 ± 33,8	905,9 ± 256,7	752,9 ± 293	823,25 ± 83,54	293,5 ± 113	755,55 ± 270	752,9 ± 293	1418 ± 150	521 ± 119,4
Thiocyanic acid, methyl ester	709	665,76 ± 0,01	153,74 ± 20,2	819,6 ± 31,24	49,07 ± 10,7	454,22 ± 53,92	37,49 ± 17,2	413,7 ± 14,1	49,07 ± 10,7	513,2 ± 33	44,09 ± 6,85
**Total**	-	**1142,37**	**835,92**	**1725,45**	**801,97**	**1277,47**	**331,01**	**1169,24**	**801,97**	**1931,67**	**565,06**
**Nitriles**											
2-Butenenitrile	621	1170,2 ± 122,3	0 ± 0	992,2 ± 92,09	0 ± 0	1043,9 ± 131,5	0 ± 0	874,74 ± 27,8	0 ± 0	1023 ± 502	0 ± 0
3-Butenenitrile	621	20,68 ± 8,16	490,41 ± 57,9	13,07 ± 1,96	564,75 ± 114	13,82 ± 2,67	484,3 ± 287	6,43 ± 1,05	564,8 ± 114	5,89 ± 1,15	775,4 ± 49,37
Butanenitrile, 3-methyl-	728	1011,8 ± 21,11	166,18 ± 42,5	858,5 ± 87,1	283,14 ± 175	663,94 ± 199,4	62,98 ± 58,4	934,64 ± 80,8	183 ± 27,2	323 ± 255	305,9 ± 29,37
Pentanenitrile	760	20,65 ± 1,85	0 ± 0	45,46 ± 1,73	0 ± 0	11,04 ± 4,17	0 ± 0	20,78 ± 2,48	0 ± 0	5,51 ± 4,45	0 ± 0
3-Pentenenitrile	768	461,8 ± 37,14	0 ± 0	294,6 ± 1,41	0 ± 0	317,02 ± 20,88	0 ± 0	275,51 ± 28	0 ± 0	453,4 ± 132	0 ± 0
Pentanenitrile, 4-methyl-	867	63,64 ± 1,28	0 ± 0	60,45 ± 9,41	0 ± 0	32,61 ± 9,18	15,98 ± 17	64,21 ± 0,98	85,94 ± 9,93	13,79 ± 12,6	125,3 ± 20,56
Hexanenitrile	876	199,6 ± 13,42	33,42 ± 11,9	194,2 ± 35,49	76,78 ± 52,7	151,17 ± 58,88	0 ± 0	190,65 ± 66,5	63,78 ± 11,6	36,89 ± 24,3	47,06 ± 2,71
Butanenitrile, 4-(methylthio)-	1092	45,6 ± 5,39	141,47 ± 35,5	205 ± 1,76	422,15 ± 87,1	334,66 ± 59,53	47,7 ± 15,6	575,58 ± 4,64	422,2 ± 87,1	173,7 ± 63,6	823,6 ± 24,7
Benzyl nitrile	1150	2402,8 ± 517,7	1008 ± 248	1757 ± 505,1	47,69 ± 5,41	3378,9 ± 608,2	1169 ± 836	2307,1 ± 25,7	47,69 ± 5,41	163 ± 48,3	51,41 ± 16,82
Octanenitrile	1185	4,28 ± 0,18	2,23 ± 0,53	3,47 ± 0,02	1,83 ± 0,81	5,02 ± 0,96	0 ± 0	5,09 ± 0,28	3,4 ± 0,74	3,43 ± 2,65	2,07 ± 0,37
Pentanenitrile, 5-(methylthio)-	1209	0 ± 0	52,42 ± 6,45	0 ± 0	96,21 ± 36,5	0 ± 0	0 ± 0	0 ± 0	96,21 ± 36,5	0 ± 0	74,17 ± 15,15
Benzenepropanenitrile	1250	375,81 ± 2,23	179,68 ± 81,4	183,3 ± 92,14	893,93 ± 190	1988,6 ± 101,7	247,6 ± 101	634,16 ± 124	893,9 ± 190	200,7 ± 95,4	345,1 ± 113,3
Methallyl cyanide		0,73 ± 0,31	184,64 ± 15,5	1,51 ± 0,68	347,71 ± 56	1,01 ± 0,77	108,3 ± 69,1	0,45 ± 0,09	347,7 ± 56	0,59 ± 0,17	276,6 ± 16,57
**Total**	-	**5777,52**	**2258,48**	**4608,80**	**2734,14**	**7941,61**	**2135,50**	**5889,30**	**2708,50**	**2403,00**	**2826,68**
**Alcohols**											
2-Buten-1-ol, (E)-	598	17,68 ± 7,09	0 ± 0	19,07 ± 0,47	0 ± 0	33,13 ± 24,93	0 ± 0	32,04 ± 22,3	752,9 ± 293	20,82 ± 4,01	0 ± 0
1-Penten-3-ol	664	103,07 ± 8,5	400,63 ± 74,2	67,54 ± 4,48	80,95 ± 46,2	63,81 ± 24,09	11,82 ± 3,48	70,26 ± 4,3	89,85 ± 53,7	74,85 ± 2,95	35,45 ± 2,15
2-Penten-1-ol, (Z)-	765	1107,2 ± 196,7	0 ± 0	618,6 ± 94,05	0 ± 0	579,27 ± 206,8	0 ± 0	729,37 ± 53,1	0 ± 0	792,7 ± 156	0 ± 0
3-Hexen-1-ol, (Z)-	859	144,97 ± 4,72	0 ± 0	45,72 ± 2,95	0 ± 0	39,12 ± 0,84	0 ± 0	157,64 ± 6,69	0 ± 0	65,97 ± 21,9	0 ± 0
2-Hexen-1-ol, (E)-	864	135,06 ± 44,37	0 ± 0	217,5 ± 154,6	0 ± 0	47,35 ± 38,75	0 ± 0	111,16 ± 32,7	0 ± 0	81,4 ± 87,4	0 ± 0
**Total**	-	**1507,98**	**400,63**	**968,46**	**80,85**	**762,67**	**11,82**	**1100,47**	**842,74**	**1035,76**	**35,45**
**Others**											
2,3-Butanedione	558	128,92 ± 9,26	101,23 ± 1,2	194,4 ± 76,67	113,32 ± 35,8	36,7 ± 9,76	7,87 ± 4,08	40,72 ± 3,11	39,76 ± 5,28	181,9 ± 81,8	141,5 ± 44,03
1-Penten-3-one	670	1365,1 ± 4,66	36,69 ± 5	311,4 ± 29,13	0 ± 0	343,08 ± 32,44	5,07 ± 3,14	326,43 ± 129	12,55 ± 5,28	185,9 ± 72,2	8,46 ± 0,78
Furan, 2-ethyl-	695	505,39 ± 57,94	397,51 ± 91	239,5 ± 66,84	79,14 ± 79,1	214,16 ± 67,67	38,59 ± 14,8	331,37 ± 50,3	66,43 ± 27,4	660,4 ± 152	105,2 ± 20,58
Furan, 2-pentyl-	996	177,1 ± 23,06	261,71 ± 37,8	178,2 ± 65,57	137,9 ± 138	346,69 ± 50,88	98,96 ± 28,2	187,99 ± 49	144,3 ± 33,7	428,5 ± 8,46	145,8 ± 9,14
3,5-Octadien-2-one, (E,E)-	1073	73,17 ± 7,92	0 ± 0	183,8 ± 4,18	0 ± 0	481,71 ± 32,9	0 ± 0	90,86 ± 10,1	0 ± 0	253,8 ± 111	0 ± 0
**Total**	-	**2249,73**	**797,14**	**1107,18**	**330,36**	**1422,34**	**150,50**	**97,7**	**263,07**	**1710,48**	**400,89**

Table S5. Compounds and their corresponding numbers used in PCA analysis (Fig. 1).

**compound**	**number in PCA plot**
Allyl Isothiocyanate	1
1-Butene, 4-isothiocyanato-	2
Isobutyl isothiocyanate	3
Benzene, (isothiocyanatomethyl)	4
2-Methylbutyl isothiocyanate	5
Benzene, (2-isothiocyanatoethyl)	6
Isopropyl isothiocyanate	7
Hexane, 1-isothiocyanato	8
Hexane, 1-isocyanato	9
Cyclopropane, isothiocyanato	10
Cyclopentyl isothiocyanate	11
Butane, 2-isothiocyanato	12
Propane, 1-isothiocyanato	13
Butane, 1-isothiocyanato	14
1-Butene, 4-isothiocyanato1-(methylthio)	15
4-Methylpentyl isothiocyanate	16
Butane, 1-isothiocyanato-3-methyl	17
Heptane, 1-isothiocyanato	18
Methane, isothiocyanato	19
Octane, 1-isothiocyanato-	20
Propane, 1-isothiocyanato-3-(methylthio)	21
n-Pentyl isothiocyanate	22
Propane, 1-isocyanato-2-methyl	23
Sulfides (di, tri, tetra, penta)	24
Thiocyanic acid, methyl ester	25
Thiophene, 2-ethyl	26
Thiophene, 2-methyl	27
Thiophene, 2-pentyl	28

S-Methyl methanethiosulphonate	29
S-Methyl pentanethioate	30
Thiocyanic acid, 2-propenyl ester	31
2-Butenal	32
Hexanal	33
2-Hexenal	34
2-Pentenal, (E)-	35
Propanal	36
2,4-Heptadienal, (E,E)	37
2-Propenal	38
NoF4-:F77nanal	39
2,4-Hexadienal, (E,E)-	40
Butanal, 2-methyl-	41
Benzeneacetaldehyde	42
3-Hexenal, (Z)-	43
2-Heptenal, (Z)-	44
2,6-Nonadienal, (E,E)-	45
2-Hexenal, (E)-	46
Benzaldehyde	47
Butanal	48
Decanal	49
Pentanal	50
Heptanal	51
Octanal	52
2-Octenal, (E)-	53
2,4-Nonadienal, (E,E)-	54
2,4-Octadienal, (E,E)-	55
2-Nonenal, (E)-	56
2-Pentenal, (E)-	57
Butanal, 3-methyl-	58
2,4-Decadienal, (E,E)-	59
Benzyl nitrile	60
2-Butenenitrile	61

Butanenitrile, 3-methyl-	62
Benzenepropanenitrile	63
Butanenitrile, 4-(methylthio)-	64
3-Pentenenitrile	65
Hexanenitrile	66
Pentanenitrile, 4-methyl-	67
Pentanenitrile	68
3-Butenenitrile	69
Octanenitrile	70
Pentanenitrile, 5-(methylthio)-	71
Hexanenitrile, 5-methyl-	72
Heptanenitrile	73
2-Penten-1-ol, (Z)-	74
2-Hexen-1-ol, (E)-	75
3-Hexen-1-ol, (Z)-	76
1-Penten-3-ol	77
2-Buten-1-ol, (E)-	78
1-hexenol	79
1-pentanol	80
1-octen-3-ol	81
1-Penten-3-one	82
Furan, 2-ethyl-	83
Furan, 2-pentyl-	84
3,5-Octadien-2-one, (E,E)-	85
